# 47例肺部恶性病变支气管内超声图像的边缘形态分析

**DOI:** 10.3779/j.issn.1009-3419.2010.05.12

**Published:** 2010-05-20

**Authors:** 正贤 陈, 静 李

**Affiliations:** 510080 广州，广东省医学科学院，广东省人民医院呼吸内科 Department of Respiratory Medicine, Guangdong General Hospital, Guangdong Academy of Medical Science, Guangzhou 510080, China

**Keywords:** 支气管内超声, 恶性病变, 边缘形态, Endobronchial ultrasound, Malignant lesion, Morphous of the edge

## Abstract

**背景与目的:**

本研究旨在通过分析支气管内超声（endobronchial ultrasound, EBUS）边缘形态的图像，探索EBUS图像的边缘形态对恶性肺部病变的诊断作用。

**方法:**

收集从2005年6月1日-2006年6月30日期间就诊的部分良恶性病例进行EBUS图像分析。

**结果:**

共确诊了47例恶性病变，另外还确诊了31例良性病变，共78例病灶良恶性诊断明确者，男56例，女22例，年龄21岁-80岁，平均（58.01±13.20）岁；78例患者的病灶部位和病变的边缘统计结果显示，不同部位和角度对超声的边缘形态没有影响；病灶的大小和病灶的边缘形态的关系分析显示病灶的大小与病灶的边缘形态无影响；恶性病变与良性病变边缘形态的比较显示，边缘清晰是恶性病变的重要表现，同时显示一定的诊断价值；根据病灶的外形与病变的性质比较，未发现超声图像的外形与病变性质的相关关系；与正常的支气管镜操作相比，EBUS的操作约进行10 min左右，未发现有关的并发症。

**结论:**

EBUS图像边缘清晰是恶性病变的一个特征，对恶性病变有一定的诊断价值。

## Abstract (en)

**Background and objective:**

By analyzing morpholoy and edge of endobronchial ultrasound (EBUS) images in 47 patients with pulmonary malignant lesions. The aim of this study is to explore the role of EBUS in the diagnosis of pulmonary malignant lesions.

**Methods:**

From June 1, 2005 to June 30, 2006, EBUS analysis was performed in a proportion of patients with malignant or benign lesions.

**Results:**

In total 78 patients of confirmed diagnosis, 47 cases were confirmed malignant lesions, and 31 were benign lesions, male 56 cases, female 22 cases, age span from 21-80 years (58.01±13.20). The statistics of the lesion edge imaging in 78 patients showed that different position and angle did not affect the morphous of the edge; analysis of the relationship between the size of the lesion and the morphous of the edge shows that the size of the lesion does not affect the morphous of the edge; the comparison between lesions of benign and malignant shows that a clear edge is the major feature of malignant lesions, indicating certain value in diagnosis; comparison between the shape of the lesion and the property of the lesion shows no relationship between them; compared to conventional bronchoscopy operations, the EBUS operation consumes approximately 10 min, and no related complication was found.

**Conclusion:**

Clear morphous of the edge in EBUS is a feature of the malignant lesion, and EBUS has certain value in the diagnosis of the malignant lesions.

支气管内超声（endobronchial ultrasound, EBUS）是将超声探头通过纤维支气管镜进入气道进行扫描成像，研究气道壁及周围组织、纵隔、肺内组织的微细结构，分析病变性质的诊断工具，由于探头外径＜2 mm，可以到达段以外的小气道，更接近病变，缩短声波路径而降低声波能量的衰减，故可采用高频技术，明显提高图像分辨力，使组织结构的成像微细化，与组织的显微结构相对应，极大地提高超声的诊断水平。故支气管内超声有“显微结构扫描”的能力，能够对支气管壁和邻近约4 cm范围内的组织结构（包括纵隔）进行高清晰度成像。将内镜的视野范围从气道腔内扩展到腔外、从大气道扩展到外周直径1.4 mm-2 mm的小气道，大大扩充了内镜医生的视野和工作范围。

基于EBUS技术的上述特点，我们对其良恶性病变的鉴别诊断进行了探索，发现不同的图像结构对良恶性病变的鉴别有不同的意义，其中一个重要的指标是EBUS图像的边缘形态，我们统计了78例患者，对其边缘形态的图像做一分析。

## 1 材料和方法

### 1.1 临床病例

从2005年6月1日-2006年6月30日期间，广东省人民医院支气管镜室连续就诊的门诊和住院患者，经胸部X线、CT检查发现肺部周围型病变，经常规可曲支气管镜（以下简称“支气管镜”）检查，明确病变位于段支气管开口以下者。全部病例最后均由病理学确诊，确诊所使用的方法包括支气管镜肺活检及细胞学检查、痰脱落细胞学检查、经皮肺穿刺活检、胸腔镜活检、开胸探查、手术切除等。所有病理学结果由两位有经验的病理科医师独立做出诊断。

### 1.2 设备

支气管镜（BF-1T30、BF-1T240或BF-1T260，日本，奥林巴斯）；腔内超声主机（ENDOECHO EU-M2000，日本，奥林巴斯），超声探头驱动器（MAJ-935，日本，奥林巴斯）；腔内超声探头（UM-BS20-26R，外径2.0 mm；UMDP20-25R，外径2.5 mm，日本，奥林巴斯）。

### 1.3 支气管内超声检查方法^[1]^

术前准备同常规支气管镜检查。先在影像资料上确定病变的所在部位，常规局部麻醉后，支气管镜进入病变所在的段支气管，通过支气管镜活检通道在管壁上注入普鲁卡因凝胶，然后经活检通道送入超声探头，直至术者感觉有阻力。如探头贴壁不佳，出现空气干扰的伪像，可注入生理盐水，或在探头外加上水囊（MH-246R，日本，奥林巴斯）。连接支气管内超声主机、探头驱动器和超声探头。驱动器旋转探头产生360o实时垂直图像。开始超声扫描，同时术者缓慢、匀速将探头往外拉出，观察超声图像。所得图像存于主机内，完成扫描后即可进行三维图像重建和显示。

### 1.4 统计学处理

采用SPSS 13.0软件分析。计量资料以Mean±SD表示，计数资料间的比较采用χ^2^检验，如四格表内期望频数＜1，或者＞1/5格子的理论频数＜5时，采用*Fisher*精确概率检验法，以*P*＜0.05为差异有统计学意义。

## 2 结果

### 2.1 病灶最后诊断及例数情况

我们共确诊了47例恶性病变，另外还确诊了31例良性病变，共78例病灶良恶性诊断明确者，男56例，女22例，年龄21岁-80岁，平均（58.01±13.20）岁，病灶最后诊断及例数见表 1。

**1:** 病灶最后诊断及例数 Final diagnosis of the lesions and No. of cases

Malignant lesions				Benign Lesions			
Final diagnosis	*n*	Morphous of the Edge		Final diagnosis	*n*	Morphous of the edge	
		Clear	Unclear			Clear	Unclar
Small cell carcinoma	3	3		Pneumonia	15	4	11
Adenocarcinoma	29	18	11	Pulmonary aspergillosis	2	2	
Squamous cell carcinoma	5	3	2	Bronchiectasis complicated with infetion	1	1	
Adenosquamous carcinoma	1		1	Pulmonary abscess	1	1	
Large cell carcinoma	1	1		Pulmonary tuberculosis	6		6
Non-small cell carcinoma	5	3	2	Wegener granulomatosis	1		1
Metastatic carcinoma of endometrium	1		1	Chondroma hamartoma	1		1
Neuroendocrine carcinoma	1	1		Penicillium marneffei pneumonia	1		1
Un-specified malignant lesions	1	1		Pulmonary interstitial Fibrosis complicated with infection	1		1
				Seborrheic pneumonia	1		1
				Inflammatory pseudotumor	1		1
Total	47	30	17	Total	31	8	23

### 2.2 患者的病灶部位和病变的边缘情况

考虑到超声的距离和角度可能对图像的影响，我们把共计78例患者的病灶部位和病变的边缘作统计，统计情况见表 2，结果显示不同部位和角度对超声的边缘形态无影响。

**2:** 病灶边界与部位的关系 Relationship between edge and position of the lesion

Edge	Position					Total
	RUL	RML	RLL	LUL	LLL	
Clear	11	3	10	5	6	35
Unclear	15	4	11	8	5	43
Total	26	7	21	13	11	78
*χ*^2^ test, *P*=0.942.						

### 2.3 病灶的大小和病灶的边缘形态的关系

表 3显示了全部病例的病灶大小和病灶边缘的关系，显示病灶的大小与病灶的边缘形态无影响。

**3:** 直径≤3 cm和直径＞3 cm病灶与边界的关系 Relationship between edge and lesion size of dia.≤3 cm and > 3 cm

Size (Diameter)	Lesion		Total	*P*
	Clear	Unclear		
All lesion				
< 3 cm	16	14	30	0.235
> 3 cm	19	29	48	
Total	35	43	78	
Malignant				
< 3 cm	15	9	24	0.846
> 3 cm	15	8	23	
Total	30	17	47	
Benign				
< 3 cm	1	5	6	0.970
> 3 cm	4	21	25	
Total	5	26	31	

### 2.4 病变性质和边缘形态的关系

通过恶性病变与良性病变边缘形态的比较显示，边缘清晰是恶性病变的重要表现，同时显示一定的诊断价值（表 4）。

**4:** 病灶性质与边界关系（例/百分比） Relationship between lesion property and edge (*n*/%)

Final diagnosis of lesions	Edge		Total
	Clear	Unclear	
Benign	5 (6.4)	26 (33.3)	31
Malignant	30 (38.5)	17 (21.8)	47
Total	42 (44.9)	36 (55.1)	78
*χ*^2^ test, *P* < 0.001. The sensitivity of diagnosis by clear lesion edge was 71.4% (30/42). Specificity was 72.2% (26/36).			

### 2.5 病灶性质与外形关系

根据病灶的外形与病变的性质比较，未发现超声图像的外形与病变性质的相关关系（表 5）。

**5:** 病灶性质与外形关系（例/百分比） Relationship between lesion property and shape (*n*/%)

Final diagnosis of lesions	Shape		Total
	Quasi-circular	Non-quasi-circular	
Benign	14(17.9)	17 (21.8)	31
Malignant	23 (29.5)	24 (30.8)	47
Total	37 (47.4)	41 (52.6)	78
*χ*^2^ test, *P*=0.746.																																														

### 2.6 EBUS相关的不良反应

与正常的支气管镜操作相比，EBUS的操作约进行10 min左右，未发现有关的并发症，普鲁卡因凝胶的使用可以达到良好的麻醉作用，同时有助提高影像的清晰度，可以推广使用。

## 3 讨论

支气管内超声应用的一个重要条件是图像的分辨率明显提高，可以把不同的组织形态清楚的和其微细结构相对应。恶性病变常有的一个清晰的边缘图像，与病变的高细胞密度有关^[2]^，尽管47例恶性病变中30例边界清晰（63.8%），但也有17例边界不清（36.2%），而良性病变也有边缘清晰的病例，两者有一定的重叠，但统计显示清楚的EBUS图像边缘仍是独立的诊断依据。

支气管内超声图像中病灶边界的病理学基础：良性肿瘤为膨胀性生长，恶性肿瘤多以膨胀性向周围快速生长^[3]^，或为覆壁（肿瘤细胞覆盖肺泡上皮细胞）生长、填充性（肺泡腔被肿瘤细胞填满）生长。周围肺组织受压产生薄层的肺萎陷带，可能是瘤体与周围肺组织界限清晰的原因之一，在超声图像上，低回声的肿瘤与含气的肺组织之间显示清晰、整齐的边界（图 1），术后大体标本也可见假包膜形成，另外肿瘤组织密度明显大于周围的肺组织，使肿瘤组织与肺组织的声阻抗差别明显，这可能也是造成边界清晰的另一原因^[4]^。

**1:**
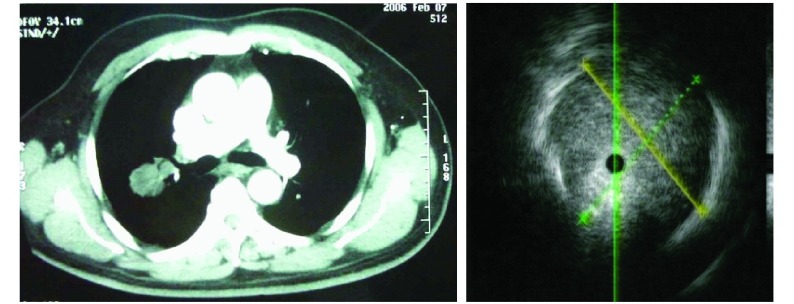
男，74岁，右B3占位，小细胞肺癌，边界清晰 Male, 74Y, Right B3 lesion, small cell lung cancer, edge was clear

17例恶性病变边界不清（36.2%），有报道^[5]^表明肺泡癌界限不清晰，同时病灶内多可见支气管气相而漏诊为良性病变或误诊。

感染灶边缘为正常含气肺实质与渗出液、炎症细胞及其碎片、肺泡萎陷、肺泡间隔机化、纤维化组织对比形成^[6]^，炎性病灶累及肺间质及肺泡壁，在扩大的过程中，由于其周围空间结构给予的阻力不同，导致向外伸展的速度不一，边界显示呈“波浪状”、“阶梯样”、“锯齿状”、“星芒状”等征象（图 2）为其特点。良性病变大多表现为边界不清。炎性假瘤多显示为边界欠清晰，肺脓疡、结核瘤等良性病变多显示为边界清晰。经活检及手术证实周围有纤维组织被膜，应引起重视。

**2:**
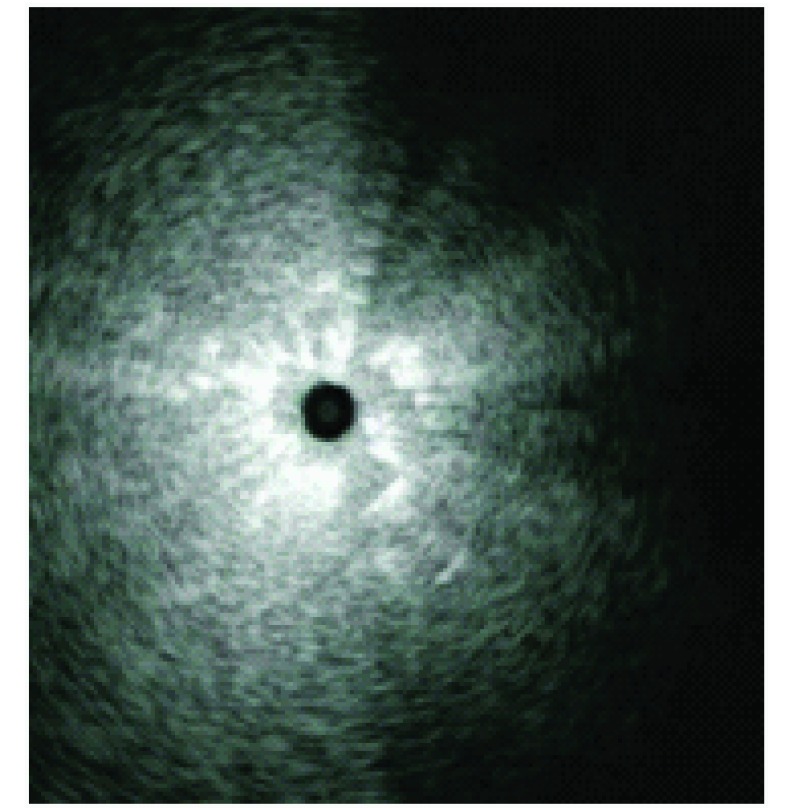
肺炎，边界不清，星芒征 Pneumonia, edge was unclear, demonstrated star sign

病灶大小和部位对边界的影响，从表 3可见，边界显示是否清晰不受病灶大小的影响，能否显示边界与病灶本身是否有边界及探头所在位置相关，当探头位于病灶内时更能清晰显示病灶边界^[7]^，有些病灶边界显示不完整，可能与20 MHz的高频探头穿透力有限有关，在条件好的情况下最大深度为5 cm，但由于声波的衰减或伪影的干扰，病灶远离探头部分往往显示不清。从表 2可见，病变部位不同对能否清晰显示病灶边界无影响。

恶性肿瘤多显示为类圆形，但肺脓疡、肺曲菌病、肺炎亦可显示为类圆型，病灶外形良恶性无明显差异。虽然形态特征对肺部周围型结节性病灶性质的判断有一定的意义，但良、恶性结节性病变的形态及边缘轮廓的特征相互交叉重叠，仅依据形态特征推断肺周围型结节性病灶的性质尚存有一定的局限性，可能与良性病变侵及肺间质向外伸展速度不均一、周围组织牵拉等存在因素有关，尤应注意结合超声影像的其它征象，综合评价诊断意义^[8]^。

尽管我们的研究显示EBUS图像边缘清晰是恶性病变的一个特征，对恶性病变有一定的诊断价值，但应用单一指标进行诊断确实存在一定的局限性，而根据多个图像特征进行综合的评价无疑有较大的意义，我科现探索使用多个指标的综合模拟诊断，与病理的符合率大大提高，如增加研究的深度，的确有望达到“显微超声扫描”的诊断目的^[9]^。
